# Establishing standardized immune phenotyping of metastatic melanoma by digital pathology

**DOI:** 10.1038/s41374-021-00653-y

**Published:** 2021-08-26

**Authors:** Bettina Sobottka, Marta Nowak, Anja Laura Frei, Martina Haberecker, Samuel Merki, Tumor Profiler consortium, Rudolf Aebersold, Melike Ak, Faisal S. Al-Quaddoomi, Jonas Albinus, Ilaria Alborelli, Sonali Andani, Per-Olof Attinger, Marina Bacac, Daniel Baumhoer, Beatrice Beck-Schimmer, Niko Beerenwinkel, Christian Beisel, Lara Bernasconi, Anne Bertolini, Bernd Bodenmiller, Ximena Bonilla, Ruben Casanova, Stéphane Chevrier, Natalia Chicherova, Maya D’Costa, Esther Danenberg, Natalie Davidson, Monica-Andreea Drăganmoch, Stefanie Engler, Martin Erkens, Katja Eschbach, Cinzia Esposito, André Fedier, Pedro Ferreira, Joanna Ficek, Bruno Frey, Sandra Goetze, Linda Grob, Gabriele Gut, Detlef Günther, Martina Haberecker, Pirmin Haeuptle, Viola Heinzelmann-Schwarz, Sylvia Herter, Rene Holtackers, Tamara Huesser, Anja Irmisch, Francis Jacob, Andrea Jacobs, Tim M. Jaeger, Katharina Jahn, Alva R. James, Philip M. Jermann, André Kahles, Abdullah Kahraman, Werner Kuebler, Jack Kuipers, Christian P. Kunze, Christian Kurzeder, Kjong-Van Lehmann, Sebastian Lugert, Gerd Maass, Markus G. Manz, Philipp Markolin, Julien Mena, Ulrike Menzel, Julian M. Metzler, Nicola Miglino, Emanuela S. Milani, Simone Muenst, Riccardo Murri, Charlotte K. Y. Ng, Stefan Nicolet, Patrick G. A. Pedrioli, Lucas Pelkmans, Salvatore Piscuoglio, Michael Prummer, Mathilde Ritter, Christian Rommel, María L. Rosano-González, Gunnar Rätsch, Natascha Santacroce, Jacobo Sarabia del Castillo, Ramona Schlenker, Petra C. Schwalie, Severin Schwan, Tobias Schär, Gabriela Senti, Franziska Singer, Sujana Sivapatham, Berend Snijder, Vipin T. Sreedharan, Stefan Stark, Daniel J. Stekhoven, Alexandre P. A. Theocharides, Tinu M. Thomas, Markus Tolnay, Vinko Tosevski, Nora C. Toussaint, Mustafa A. Tuncel, Marina Tusup, Audrey Van Drogen, Marcus Vetter, Tatjana Vlajnic, Sandra Weber, Walter P. Weber, Rebekka Wegmann, Michael Weller, Fabian Wendt, Norbert Wey, Andreas Wicki, Mattheus HE Wildschut, Bernd Wollscheid, Shuqing Yu, Johanna Ziegler, Marc Zimmermann, Martin Zoche, Gregor Zuend, Mitchell P. Levesque, Reinhard Dummer, Holger Moch, Viktor Hendrik Koelzer

**Affiliations:** 1 Department of Pathology and Molecular Pathology, University and University Hospital Zurich, Zurich, Switzerland; 2 Department of Dermatology, University and University Hospital Zurich, Zurich, Switzerland; 3 ETH Zurich, Department of Biology, Institute of Molecular Systems Biology, Otto-Stern-Weg 3, 8093 Zurich, Switzerland; 4 University Hospital Zurich, Department of Dermatology, Gloriastrasse 31, 8091 Zurich, Switzerland; 5 ETH Zurich, NEXUS Personalized Health Technologies, John-von-Neumann-Weg 9, 8093 Zurich, Switzerland; 6 SIB Swiss Institute of Bioinformatics, Lausanne, Switzerland; 7 ETH Zurich, Department of Health Sciences and Technology, Otto-Stern-Weg 3, 8093 Zurich, Switzerland; 8 University Hospital Basel, Institute of Medical Genetics and Pathology, Schönbeinstrasse 40, 4031 Basel, Switzerland; 9 ETH Zurich, Department of Computer Science, Institute of Machine Learning, Universitätstrasse 6, 8092 Zurich, Switzerland; 10 University Hospital Zurich, Biomedical Informatics, Schmelzbergstrasse 26, 8006 Zurich, Switzerland; 11 University Hospital Zurich, Department of Pathology and Molecular Pathology, Schmelzbergstrasse 12, 8091 Zurich, Switzerland; 12 F. Hoffmann-La Roche Ltd, Grenzacherstrasse 124, 4070 Basel, Switzerland; 13 Roche Pharmaceutical Research and Early Development, Roche Innovation Center Zurich, Wagistrasse 10, 8952 Schlieren, Switzerland; 14 University of Zurich, VP Medicine, Künstlergasse 15, 8001 Zurich, Switzerland; 15 ETH Zurich, Department of Biosystems Science and Engineering, Mattenstrasse 26, 4058 Basel, Switzerland; 16 University Hospital Zurich, Clinical Trials Center, Rämistrasse 100, 8091 Zurich, Switzerland; 17 ETH Zurich, Institute of Molecular Health Sciences, Otto-Stern-Weg 7, 8093 Zurich, Switzerland; 18 University of Zurich, Department of Quantitative Biomedicine, Winterthurerstrasse 190, 8057 Zurich, Switzerland; 19 University of Zurich, Institute of Molecular Life Sciences, Winterthurerstrasse 190, 8057 Zurich, Switzerland; 20 Roche Pharmaceutical Research and Early Development, Roche Innovation Center Basel, Grenzacherstrasse 124, 4070 Basel, Switzerland; 21 University Hospital Basel and University of Basel, Department of Biomedicine, Hebelstrasse 20, 4031 Basel, Switzerland; 22 Roche Diagnostics GmbH, Nonnenwald 2, 82377 Penzberg, Germany; 23 ETH Zurich, Department of Chemistry and Applied Biosciences, Vladimir-Prelog-Weg 1-5/10, 8093 Zurich, Switzerland; 24 Cantonal Hospital Baselland, Medical University Clinic, Rheinstrasse 26, 4410 Liestal, Switzerland; 25 University Hospital Basel, Gynecological Cancer Center, Spitalstrasse 21, 4031 Basel, Switzerland; 26 University Hospital Basel, Spitalstrasse 21/Petersgraben 4, 4031 Basel, Switzerland; 27 University Hospital Basel, Department of Information- and Communication Technology, Spitalstrasse 26, 4031 Basel, Switzerland; 28 University Hospital Basel, Brustzentrum, Spitalstrasse 21, 4031 Basel, Switzerland; 29 University Hospital Zurich, Department of Medical Oncology and Hematology, Rämistrasse 100, 8091 Zurich, Switzerland; 30 University Hospital Zurich, Department of Gynecology, Frauenklinikstrasse 10, 8091 Zurich, Switzerland; 31 University of Zurich, Services and Support for Science IT, Winterthurerstrasse 190, 8057 Zurich, Switzerland; 32 University of Bern, Department of BioMedical Research, Murtenstrasse 35, 3008 Bern, Switzerland; 33 ETH Zurich, Department of Biology, Wolfgang-Pauli-Strasse 27, 8093 Zurich, Switzerland; 34 Roche Pharmaceutical Research and Early Development, Roche Innovation Center Munich, Roche Diagnostics GmbH, Nonnenwald 2, 82377 Penzberg, Germany; 35 University Hospital Basel, Brustzentrum & Tumorzentrum, Petersgraben 4, 4031 Basel, Switzerland; 36 University Hospital Basel and University of Basel, Department of Surgery, Brustzentrum, Spitalstrasse 21, 4031 Basel, Switzerland; 37 University Hospital and University of Zurich, Department of Neurology, Frauenklinikstrasse 26, 8091 Zurich, Switzerland; 38 University of Zurich, Faculty of Medicine, Zurich, Switzerland; 39 University Hospital Zurich, Rämistrasse 100, 8091 Zurich, Switzerland

**Keywords:** Imaging the immune system, Melanoma, Predictive markers

## Abstract

CD8+ tumor-infiltrating T cells can be regarded as one of the most relevant predictive biomarkers in immune-oncology. Highly infiltrated tumors, referred to as inflamed (clinically “hot”), show the most favorable response to immune checkpoint inhibitors in contrast to tumors with a scarce immune infiltrate called immune desert or excluded (clinically “cold”). Nevertheless, quantitative and reproducible methods examining their prevalence within tumors are lacking. We therefore established a computational diagnostic algorithm to quantitatively measure spatial densities of tumor-infiltrating CD8+ T cells by digital pathology within the three known tumor compartments as recommended by the International Immuno-Oncology Biomarker Working Group in 116 prospective metastatic melanomas of the Swiss Tumor Profiler cohort. Workflow robustness was confirmed in 33 samples of an independent retrospective validation cohort. The introduction of the intratumoral tumor center compartment proved to be most relevant for establishing an immune diagnosis in metastatic disease, independent of metastatic site. Cut-off values for reproducible classification were defined and successfully assigned densities into the respective immune diagnostic category in the validation cohort with high sensitivity, specificity, and precision. We provide a robust diagnostic algorithm based on intratumoral and stromal CD8+ T-cell densities in the tumor center compartment that translates spatial densities of tumor-infiltrating CD8+ T cells into the clinically relevant immune diagnostic categories “inflamed”, “excluded”, and “desert”. The consideration of the intratumoral tumor center compartment allows immune phenotyping in the clinically highly relevant setting of metastatic lesions, even if the invasive margin compartment is not captured in biopsy material.

## Abstract (web summary)

The authors present a robust diagnostic algorithm based on digital pathology and image analysis that quantifies intratumoral and stromal CD8+ T-cell densities in the tumor center and invasive margin compartment in metastatic melanoma. Spatial CD8+ T-cell densities are translated into the clinically relevant immune diagnostic categories “inflamed”, “excluded”, and “desert”. Their approach also allows efficient immune phenotyping of metastatic lesions, on biopsy material or even in the absence of material from the invasive margin.

## Introduction

Melanoma is one of the prime examples for the success of immune checkpoint inhibition in the clinic. Survival of metastatic melanoma patients improved from 9 months before 2011 to currently >3 years^1–3^. Nevertheless, secondary resistance after a primary response is observed in about 40% of metastatic melanoma patients^4,5^ but robust predictive biomarkers to inform clinical decision-making on immunotherapy are missing.

Clinical evidence from a variety of malignancies—including melanoma—suggest that the success of immune checkpoint inhibitors relies critically on preexisting tumor infiltrative CD8+ T cells^6,7^. Proportionally to their presence, tumors can broadly be categorized into inflamed and non-inflamed^8,9^. Inflamed tumors are characterized by high CD8+ T-cell densities and reflect preexisting immunity, whereas non-inflamed tumors are immunologically ignorant with only scarce CD8+ T cells. The functional relevance of these categorizes was elucidated by clinical studies examining their amount and distribution in serial on-treatment biopsies^6^. Three distinct distribution patterns of CD8+ T cells were identified: (1) high densities of intraepithelial CD8+ T cells corresponding to inflamed tumors associated with a favorable response to immune checkpoint inhibition; (2) a poor infiltrate corresponding to non-inflamed/desert tumors, or (3) high densities of CD8+ T cells at the tumor margin without tumor infiltration, referred to as immune excluded^6,10^. Both non-inflamed/desert and excluded immune phenotypes were strongly correlated with non-response to immune checkpoint inhibitors in the clinic. These three distribution patterns were therefore translated into clinically relevant categories “hot” corresponding to inflamed and “cold” showing either an immune desert or excluded pattern^11^. CD8+ T cells represent the currently most actionable target of immune checkpoint inhibitors^12^. Their high baseline prevalence in the intraepithelial compartment can be regarded as a strong predictor to immune checkpoint inhibition. Yet, neither for melanoma nor for any other entity are tumor-infiltrating CD8+ T cells currently assessed in routine diagnostic practice despite their essential role to predict immunotherapy success.

Pathologists perform tissue diagnostics for virtually all cancer patients. Additional immunohistochemistry is carried out on a routine basis and is available in every pathology institute. Visual scoring of tumor-infiltrating T cells however suffers from inter-observer variability and poor reproducibility^13,14^. Computational scoring by digital pathology is regarded as superior, especially if board-certified pathologists verify image segmentation and cell detection^14^. We therefore established a quantitative, easy to implement and robust approach considering state-of-the-art concepts^14,15^. Importantly, we did not only quantify CD8+ tumor-infiltrating T cells densities within the respective tumor compartments, but translated their densities into an applicable clinical immune diagnosis. By applying pathologist-trained deep learning algorithms to diagnostic immunohistochemistry stains, we were able to subdivide the tumor center compartment into an intratumoral cellular and intratumoral stromal compartment for accurate measurement of CD8+ T-cell spatial distribution. We assessed the invasive margin as recommended by the International Immuno-Oncology Biomarker Working Group^14,15^ but discovered it not be mandatory for our proposed immune diagnostic algorithm in the assessment of metastatic lesions. Based on the Swiss Tumor Profiler discovery cohort^16^ we identified CD8+ T-cell density cut-offs to classify melanomas for each immune diagnostic category. We verified our proposed diagnostic algorithm in an independent retrospective melanoma validation cohort and show significant correlations between the pathologists’ and the digital pathology based immune diagnosis.

The combination of immunohistochemistry, image segmentation, deep learning, and quality control by pathologists yielded high-quality results with robust and reproducible findings in two independent cohorts of metastatic melanoma lesions. The targeted investigation of the intratumoral cellular compartment allowed reproducible immune phenotyping in metastatic sites, clinically most relevant after excision of the primary tumor but often biopsied without the invasive margin compartment. Our study therefore provides a novel diagnostic dimension by categorizing quantitative spatial data on CD8+ T cells into a functional and clinically relevant immune phenotype and may become an additional predictive immuno-oncology tool.

## Materials and methods

### Patient cohorts: discovery and validation cohort

The Swiss Tumor Profiler is an approved, observational clinical study (Registration IDs: 2018-02050 (KEK ZH, Switzerland), 2018-02052 (EKNZ, Basel, Switzerland), 2019-01326 (KEK ZH, Switzerland)) with the intention to identify novel treatment targets in prospectively collected tumor patient samples using cutting-edge technologies^16^. In the past 2 years, 116 melanoma patients were enrolled in the Tumor Profiler study. Of these, an additional longitudinal sample was available from ten patients, resulting in a total of 126 samples (Table 1). Inclusion criteria for the present study were the availability of a high-quality digital scan of a CD8 immunohistochemistry stain, and serial section H&E for pathology review. Five samples were excluded due to unrecoverable pre-analytical issues. In detail, one case revealed repeatedly unspecific staining or poor nuclear morphology, and four melanomas displayed a spindle cell morphology only. Seven additional melanomas showed two distinct but adjacent immune phenotypes within one metastatic location by pathology review, which we classified as lesions with a “dual immune phenotype”. These cases were evaluated, but are not subject of the here presented study and will be published separately. Metastatic sites included lymph node metastases but also distant metastases in brain, soft tissue and different anatomical locations like pleura, lung, and intestine summarized as “other” metastatic sites (Table 1). In the present study, the Tumor Profiler melanoma samples were investigated as the discovery cohort. For the validation cohort, we searched for melanoma metastases in lymph node, brain, and soft tissue in the archives of the Department of Pathology and Molecular Pathology, University Hospital Zurich between 2013 and 2018. The validation cohort (BASEC: 2018-02282) comprised in total 33 samples of 28 patients (Supplementary Table 1).

**Table 1:** Discovery cohort: clinicopathological data of the Tumor Profiler Melanoma cohort used as the discovery cohort.

Clinicopathological parameters patients (*n* = 116)	Site of metastasis (*n* = 126)							
	Brain (*n* = 15)		Soft tissue (*n* = 59)		LN (*n* = 37)		Other (*n* = 15)	
	No.	%	No.	%	No.	%	No.	%
Gender								
F	7	46.7	28	47.5	12	32.4	5	33.3
M	8	53.3	31	52.5	25	67.6	10	66.7
Age (years)								
<40	2	13.3	3	5.1	2	5.4	2	13.3
40–49	3	20.0	2	3.4	2	5.4	5	33.3
50–59	6	40.0	16	27.1	13	35.1	1	6.7
60–69	1	6.7	16	27.1	8	21.6	2	13.3
70–79	2	13.3	19	32.2	8	21.6	5	33.3
≥80	1	6.7	3	5.1	4	10.8	0	0
Histological subtype								
Cutaneous	12	80.0	43	72.9	31	83.8	10	66.7
Mucosal	0	0.0	3	5.1	1	2.7	3	20.0
Ocular	1	6.7	8	13.6	0	0.0	2	13.3
Unknown primary	2	13.3	5	8.5	5	13.5	0	0.0
Stage								
III	0	0.0	13	22.0	19	51.4	1	6.7
IV	15	100.0	46	78.0	18	48.6	14	93.3
Immune diagnosis								
Desert	7	46.7	19	32.2	8	21.6	2	13.3
Excluded	5	33.3	26	44.1	21	56.8	10	66.7
Inflamed	2	13.3	10	16.9	6	16.2	3	20.0
Dual phenotype	1	6.7	4	6.8	2	5.4	0	0.0

126 samples from different anatomical metastatic sites were collected from 116 patients.

### Tissue selection, immunohistochemistry, and digitalization

After tissue sampling according to the Tumor Profiler guidelines, melanoma metastases were formalin-fixed and paraffin-embedded for routine pathology assessment. Whole slide sections were cut at 2 μm and stained by immunohistochemistry on an automated immunostainer (Ventana Medical Systems, Tucson, AZ, USA) utilizing the monoclonal rabbit-anti-human CD8 (Ventana Medical Systems, clone SP57, dilution 1:100) with pretreatments according to the manufactures’ instructions. Antibody detection was performed using the ultraView Universal Alkaline Phosphatase Red Detection Kit (Ventana Medical Systems). Stained slides were digitalized at ×40 magnification and a resolution of 0.25 µm/pixel using the Ventana DP200 slide scanner (Ventana Medical Systems). Accuracy of tissue sections, immunohistochemistry stains and scans was independently controlled by two pathologists (B.S. and V.H.K.) and a digital imaging expert (M.N.).

### Visual scoring and tumor compartments

Two pathologists (B.S. and V.H.K.) semi-quantitatively evaluated CD8+ tumor-infiltrating T cells according to the state-of-the-art recommendations by the International Immuno-Oncology Biomarker Working Group including the tumor compartments tumor center and invasive margin^14,15^. In addition, the “tumor center” compartment was refined into an intratumoral and stromal compartment as previously suggested^17^. The (1) intratumoral tumor center compartment was defined as the intratumoral cellular compartment of the tumor consisting of tumor cells without intervening intratumoral stroma; the (2) stromal tumor center compartment was defined as intratumoral stroma without tumor cells. In lymph nodes, preexisting lymphatic tissue was excluded from analysis. For all cases, whole slides were evaluated to capture CD8+ T-cell heterogeneity and accurately assess T-cell infiltrates in the intratumoral/tumor melanocytic compartment^15^.

### CD8+ T-cell immune phenotypes

To translate the spatial distribution pattern of tumor-infiltrating CD8+ T cells into an immune phenotype, tumors were defined as (1) immune desert if only very rare and isolated CD8+ T cells could be detected in any of the assessed tumor compartments, (2) immune excluded if CD8+ T cells had arrived at the tumor environment but could only be found at the invasive margin or within the stroma and only rare and isolated T cells were present in the intratumoral compartment, and (3) inflamed if CD8+ T cells could be detected in the stromal compartment but, importantly, infiltrated the tumor parenchyma and displayed direct contact with tumor cells as previously suggested^17^. Immune phenotypes were re-assessed by two pathologists (B.S., V.H.K.) after a wash-out period of >4 weeks.

### Digital pathology

For digital immune phenotyping, we developed an end-to-end image analysis pipeline in the HALO^AI^ platform consisting of (1) expert pathologist review and annotation of tumor center and invasive border according to the recommendations of the International Immuno-Oncology Biomarker Working Group, followed by automated (2) deep learning-based tissue classification with (3) cell segmentation, and (4) spatially resolved detection and scoring of CD8+ T-cell infiltrates as visualized by immunohistochemistry stains to achieve highly accurate and automated differentiation of immune cell infiltrates in each case. At baseline, we utilized pathologist-defined annotations of cancer tissue (*n* = 333 separate regions, 146.16 mm^2^), desmoplastic stroma (*n* = 77; 4.88 mm^2^), immune-infiltrated stroma (*n* = 147; 8.45 mm^2^), glass background (*n* = 37; 0.73 mm^2^), pigment deposition (*n* = 87; 8.73 mm^2^), and areas of hemorrhage and necrosis (*n* = 52; 2.45 mm^2^) for a total of *n* = 733 separate tissue regions with a total area of 171.4 mm^2^ as ground truth dataset for training a deep neural network (Densenet^18^, patch size: 256 × 256 pixels) for tissue segmentation. Classification outputs were visualized as mark-up images (Supplementary Fig. 2) and provided to expert pathologists for corrections in an active learning process. Performance was cross-validated against ground truth annotations on the unseen dataset of the validation cohort. For cell-level analysis, nuclei were segmented using a seeded watershed on the hematoxylin counterstain followed by cell/nuclear boundary detection and post processing according to pathologist-controlled cellular parameters, such as nuclear size, roundness, and optical density. Marker positivity (Alkaline Phosphatase staining) was detected and analyzed according to pathologist-set positivity thresholds. CD8+ T cells were quantified separately in each tumor region and tissue compartment and normalized by area (Supplementary Fig. 1) to obtain CD8+ T-cell densities per tumor compartment.

### Diagnostic accuracy and statistics

Diagnostic accuracy of cut-off values was evaluated according to established standards^19^. Assessment of differences between CD8+ T-cell densities among tumor compartments and among immune diagnoses was performed by multiple comparisons for two-way ANOVA using the Holm–Šídák posttest. All statistical analyses including correlation analysis and linear regression were conducted using GraphPad Prism (version 8.0).

## Results

**Fig. 1:**
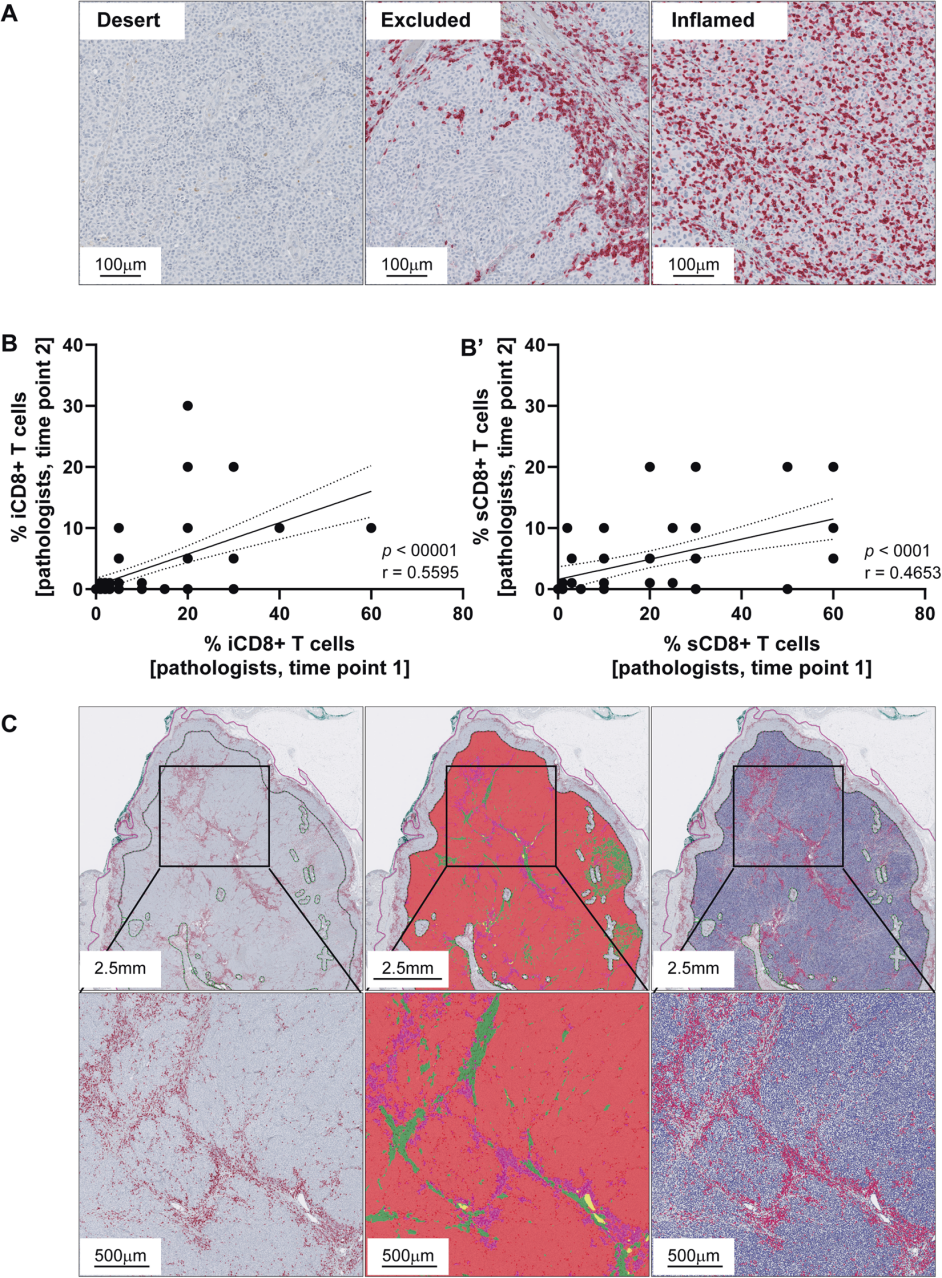
Illustration of CD8+ T cells in relation to their immune phenotypes. (**A**) Absence of T cells in immune desert tumors, accumulation of T cells at the invasive margin or in the intratumoral stroma without effective invasion in immune excluded tumors and infiltration of T cellsinto the tumor parenchyma in inflamed tumors. Correlation analysis of pathologists’ first and second semi-quantitative evaluation of the intratumoral CD8+ (iCD8+) T-cell (**B**) and stromal CD8+ (sCD8+) T-cell (**B**′) percentages; solid lines = best fit, dotted lines = error bars. Pathologist guided annotation (**C**) of the tumor border with 1 mm invasive margin (**C**, left) with exclusion of artefacts according to the recommendations by the working group. AI-based segmentation (**C**, middle) of melanoma metastases into tumor (red), inflamed stroma (purple), and desmoplastic stroma (green); exclusion of glass background, melanin pigment, hemorrhage, and necrosis. Cell segmentation andscoring at single cell resolution (**C**, right) evaluating CD8+ infiltration per μm2 Q9 in each compartment and tissue type (Color figure online).

One hundred sixteen melanoma patients were enrolled in the Swiss Tumor Profiler study^16^ between 2019 and 2020. An additional longitudinal sample was available for ten patients, resulting in a total of 126 tissue samples from lymph node, brain, soft tissue, and different anatomical locations such as pleura, lung, intestine summarized as “other” metastatic sites (Table 1). A total of 114 metastatic samples met the inclusion criteria for the current study and were examined. CD8 immunohistochemistry of whole tissue sections revealed the spatial distribution of CD8+ tumor-infiltrating T cells (Fig. 1A) depicted according to the previously suggested immune phenotypes^10^. Inter- and intraobserver variability of the intratumoral CD8+ (iCD8+) T-cell- (Fig. 1B) and stromal CD8+ (sCD8+) T-cell- (Fig. 1B′) related immune phenotypes between pathologists was lower than expected and improved upon re-assessment^13,20^. For computational CD8+ T-cell assessment, the invasive margin and tumor center region were annotated on digital whole slide images and areas with relevant artefacts were excluded (Fig. 1C and Supplementary Fig. 2). An average tissue area of 73 mm^2^ (range: 32 mm^2^ (average area brain samples)–100 mm^2^ (average area lymph node samples)) was analyzed per case and an average of 3.3 × 10^4^ CD8+ T cells (range: 6.8 × 10^3^ (average CD8+ cells brain samples)–6.5 × 10^4^ (average CD8+ cells lymph node samples)) were detected and classified for immune phenotyping (Supplementary Fig. 1). Tertiary lymphoid structures, which are regarded as confounders in the assessment of tumor-infiltrating T cells, were excluded according to established guidelines by the International Immuno-Oncology Biomarker Working Group^14^. Detection of CD8+ T cells within the regions of interest was performed using digital image analysis as described in “Methods” (Fig. 1C and Supplementary Fig. 2) and CD8+ T-cell infiltration densities per μm^2^ tissue area were derived for each tumor compartment.

**Fig. 2:**
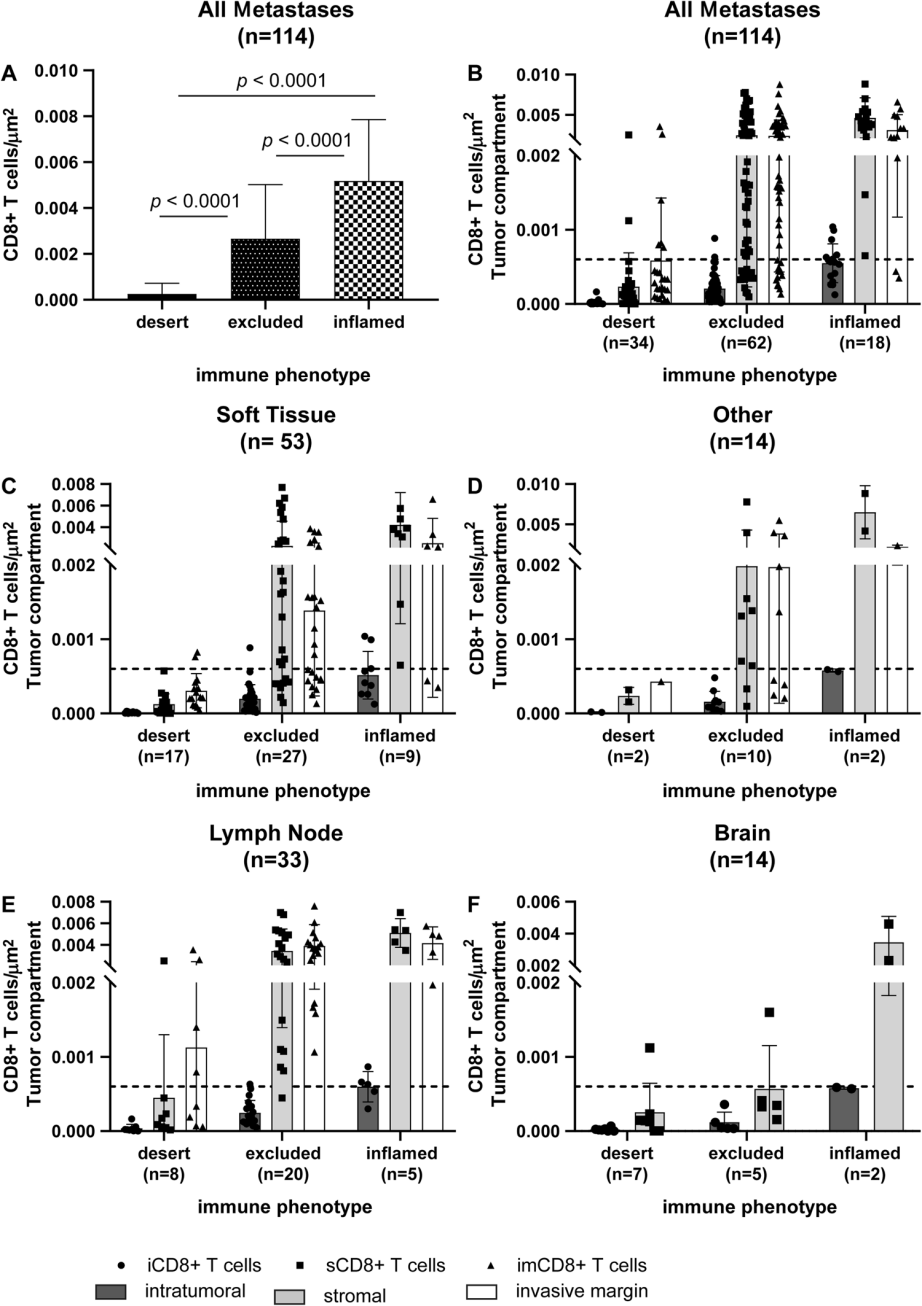
Discovery cohort. Densities of CD8+ T cells/μm^2^ in the discovery cohort independent of the tumor compartment (**A**). Total densities of CD8+ T cells differed significantly between immune desert, excluded, and inflamed tumors (**A**). Densities of CD8+ T cells/μm^2^ depicted according to their spatial distribution among the tumor compartments intratumoral (iCD8+ T cells; left; dark gray columns), stroma (sCD8+ T cells; middle; light gray columns), and invasive margin (imCD8+ T cells; right; white columns) in relation to the immune diagnosis and site of metastasis (**B**–**F**); line at 0.06 CD8+ T cells/μm^2^.

**Fig. 3:**
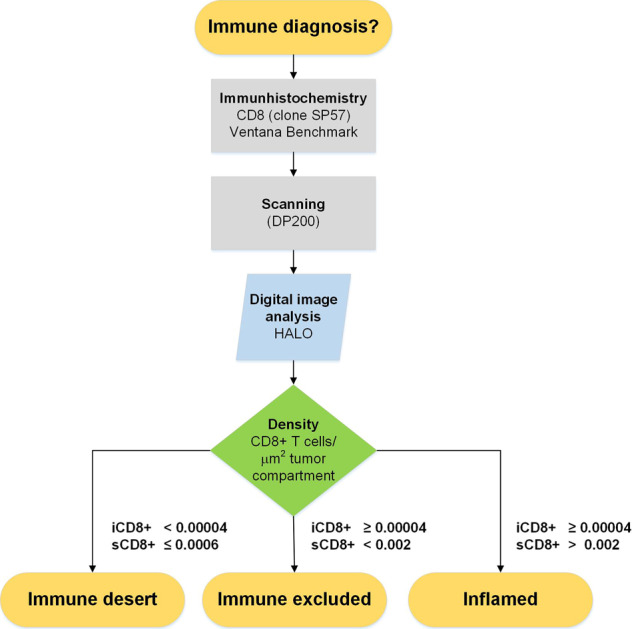
Diagnostic algorithm. Proposed diagnostic algorithm using a highly standardized and reproducible approach to define an immune diagnosis based on densities of tumor-infiltrating CD8+ T cells/μm^2^/ tumor compartment.

Overall, CD8+ T-cell densities were significantly lower in tumors classified as immune desert than as excluded or inflamed by expert pathologist review (Fig. 2A) endorsing the semi-quantitative and categorical evaluation by light microscopy. Further sub-classification of CD8+ T-cell densities per tumor compartment confirmed their differential spatial distribution among immune phenotypes in all assessed samples (Fig. 2B) and irrespective of the anatomical site of metastasis (Fig. 2C–F). According to the observed means (Table 2A) and significances (Table 2B) in the discovery cohort, a diagnostic algorithm emerged using a combination of intratumoral (iCD8) and stromal (sCD8) T-cell densities as cut-offs values to categorize the measured findings into the correct immune diagnostic category (Fig. 3). All tumors were successfully categorized into the category (1) desert if either iCD8+ and/or sCD8+ fulfilled the cut-off values, (2) excluded, or (3) inflamed if the respective iCD8+ and sCD8+ cut-off values were fulfilled.

**Table 2:** Discovery cohort: means of CD8+ T-cell densities according to their spatial distribution and immune phenotype within the discovery cohort (A) and the corresponding *p* values using the Holm–Šídák method for multiple comparisons (B).

A			
Mean CD8+ T cells/μm^2^	Immune phenotypes		
	Desert (*n* = 34)	Excluded (*n* = 62)	Inflamed (*n* = 18)
Tumor compartments			
iCD8+	0.00002	0.0002	0.0006
sCD8+	0.0002	0.002	0.005
imCD8+	0.0006	0.002	0.003

B			
*p* values	Immune phenotypes		
	Desert vs. excluded	Desert vs. inflamed	Excluded vs. inflamed
Tumor compartments			
iCD8+	ns	<0.05	ns
sCD8+	<0.0001	<0.0001	<0.01
imCD8+	<0.0001	<0.0001	ns

Invasive margin (im) CD8+ for desert *n* = 24, excluded *n* = 54, and inflamed *n* = 13.

**Fig. 4:**
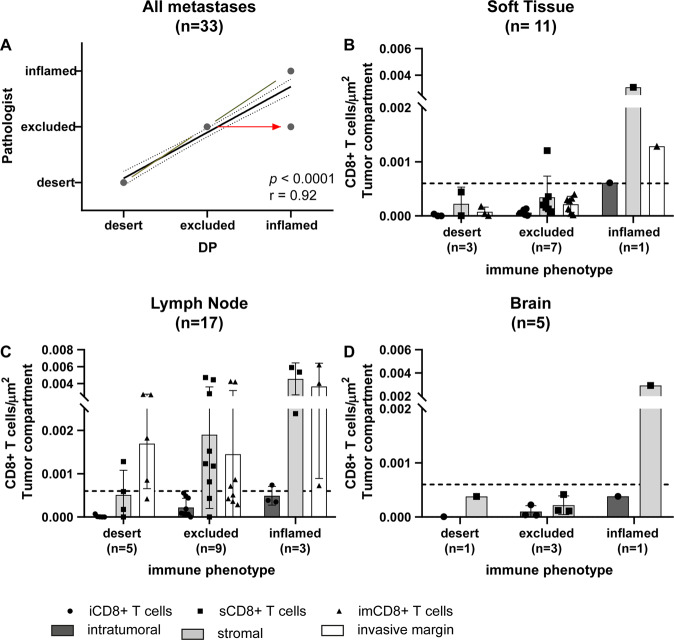
Validation cohort. Linear regression and correlation analysis between the digitally assessed and classified immune diagnoses and the pathologists’ diagnoses in the validation cohort (**A**). While all cases from the diagnoses “desert” and “inflamed” grouped to their appropriate category, three cases from the diagnoses “excluded” clustered to “inflamed” (red arrow) when applying the suggested algorithm. The black line shows the measured linear regression line with the 95% confidence interval, the green line depicts the perfect fit (**A**). Densities of CD8+ T cells in the validation cohort according to their spatial distribution among the tumor compartments intratumoral (iCD8+ T cells; left; dark gray columns), stroma (sCD8+ T cells; middle; light gray columns), and invasive margin (imCD8+ T cells; right; white columns) in relation to the digital-based immune diagnosis and site of metastasis (**B**–**D**); line at 0.06 CD8+ T cells/μm^2^ (Color figure online).

The assessment of the invasive margin was found not to be essential for establishing a digital immune diagnosis and was therefore omitted. This allowed the evaluation of metastatic biopsy material, which often lacks a sample of the invasive margin as we also observed in 23 cases of the discovery cohort (Fig. 2). To validate our proposed classification algorithm, we established a retrospective cohort of melanoma metastases from lymph node, brain, and soft tissue sites (Supplementary Table 1). Tumor tissue was treated in the same pre-analytical and analytical fashion including scoring by pathologists. Clustering of measured CD8+ T-cell densities into one of the three immune diagnoses according to the proposed diagnostic algorithm (Fig. 3) correlated significantly with the pathologists’ judgment (Fig. 4A). To assess the diagnostic performance of our suggested algorithm, we calculated a confusion matrix using the pathologists immune diagnostic category as “gold standard” or “ground truth” indicated as “pathologist diagnosis” (Table 3). A precision of sensitivity of 100% was reached for classification of tumors in the desert category with a few excluded tumors (sensitivity 83%, specificity 100%) assigned to the inflamed immune diagnostic category (sensitivity 100%, specificity 62.5%) (Table 3). In-depth analysis of the T-cell infiltration patterns revealed a similar spatial distribution and comparable densities of CD8+ T cells as in the development cohort (Fig. 4B–D and Table 4). All tumors were successfully classified according to the established criteria (Supplementary Table 2). Taken together, both the pre-analytical and analytical workflows could be robustly confirmed in an independent validation cohort endorsing the here proposed diagnostic algorithm to establish an immune diagnosis by digital pathology.

**Table 3:** The depicted confusion matrix illustrates the performance of our proposed diagnostic algorithm to translate measured CD8+ T-cell densities into a respective immune diagnostic category in the validation cohort.

Confusion matrix	Pathologist diagnosis			True positives (TP)	True negatives (TN)	False positives (FP)	False negatives (FN)	Sensitivity (Recall)	Specificity	Positive predictive value (Precision)
	Desert (*n* = 10)	Excluded (*n* = 18)	Inflamed (*n* = 5)							
Predicted										
Desert	10	0	0	10	23	0	0	100%	100%	100%
Excluded	0	15	0	15	15	0	3	83%	100%	100%
Inflamed	0	3	5	5	25	3	0	100%	89%	62.5%

Whereas the prediction, sensitivity, and specificity of immune desert tumors was very robust, few excluded tumors were attributed to the inflamed immune diagnostic category.

## Discussion

We here report one the first and most comprehensive computational assessments of CD8+ tumor-infiltrating T cells in clinical samples of metastatic melanoma. The intention is to provide a practical and robust diagnostic algorithm that can be implemented into a routine pathology workflow with direct clinical relevance. The focus on CD8+ T cells is in line with the notion that CD8+ T cells represent currently one of the most actionable targets of immune checkpoint inhibitors as also observed in anti-PD1 treated metastatic melanoma patients^21^.

As described for melanoma and other malignancies^22,23^, evaluation of the spatial distribution of CD8+ T cells proved to be an essential diagnostic criterion rather than assessment of the mere absolute counts or densities. Indeed, digital image-based approaches using immunohistochemistry^24,25^ or multispectral^26^ analysis recently demonstrated the clinical significance of spatial CD8+ T-cell evaluation in metastatic melanoma predicting response to BRAF or MAPK inhibition and correlating with improved response to immunotherapy. The introduction of the intratumoral tumor center compartment into our analysis was identified as a crucial factor to establish an immune diagnosis in metastatic disease. While this compartment is often omitted mostly due to technical reasons by not utilizing immunohistochemistry for tumor-infiltrating lymphocyte detection^14,15^, intratumoral CD8+ T cells were described to be prognostic in melanoma^27^ providing a clear rationale for their assessment. The combined evaluation of intratumoral and stromal CD8+ T cells within the tumor center compartment correlated well with the immune diagnoses rendered by expert pathologists using light microscopy. Additionally, densities of intratumoral CD8+ T cells emerged to be more relevant than invasive margin CD8+ T cells to render an immune diagnosis, thereby providing the proof of principle for a reproducible assessment method of metastatic biopsy material that often lacks tissue from the invasive margin.

Our approach respects all concerns of the current state-of-the-art recommendations^14^ and avoids methods requiring immunofluorescence that are less robust and less frequently established in diagnostic pathology labs^21^. The here proposed diagnostic algorithm is based on a robust pre-analytical workflow and exclusively uses highly standardized devices in an approved diagnostic environment. The algorithmically derived immune diagnoses rely on absolute density measurements of intratumoral and stromal CD8+ T cells within the tumor center compartment. Image-based analytic approaches can help to overcome subjectivity in the assessment of slide-based prognostic and predictive indicators and have potential to replace the currently employed light-microscopic methods in the clinic^28,29^.

Despite these advantages, our approach still shows limitations. Quality control of pre-analytical and analytical workflows by board-certified pathologists was essential and remains indispensable. While a complete hands-off end-to-end pipeline for immune phenotyping can be envisaged, this is not supported by current medical regulations. Expert review is expected to remain a key component of digital image analysis algorithms, which will progressively simplify and enhance the pathologist interpretation of key prognostic and predictive biomarkers. Key areas for development are underlined by some of the challenges seen in the present cohort. In particular, lymph node metastases represent the most challenging anatomical location due to the difficulty to clearly distinguish between preexisting lymphoid stroma and true tumor-associated stroma, which is true for both visual and digital scoring. Also, unusual morphologies like melanoma with spindle cell features will require further expansion of the training cohort using additionally collected rare morphologies to achieve generalizability of the proposed algorithm to these rare cases. Last, the here proposed cut-off values were empirically defined and lack clinical validation. Nevertheless, the classification accuracy of our proposed diagnostic algorithm was promising, and calls for validation in a larger, well-characterized prospective cohort according to the state-of-the-art recommendations^19^ ideally in consideration of its clinical utility and preferably in various tumor entities investigating treatment-naïve samples with appropriate follow-up.

While in-depth immune phenotyping by highly multiplexed visualization methods or expression profiling of the tumor immune microenvironment certainly represent key methods to understanding entire immune landscapes, they require challenging pre-analytics, are still exploratory, costly and currently restricted to a few centers due to technical limitations. Currently established immune-oncology biomarkers like the tumor mutational burden or PDL1 expression^30^ may give insight into neo-antigenicity or a preexisting immune response but are expensive and remain uninformative regarding the quantity and spatial distribution of tumor-infiltrating CD8+ T cells, one of the key prerequisites for successful response to immunotherapy.

In conclusion, our study underscores the importance of a spatial assessment of tumor-infiltrating CD8+ T cells. This information can only be derived from tissue sections and is currently impossible to reliably evaluate by bulk methods. Our study hereby provides an additional diagnostic possibility for immunotherapy biomarkers in precision medicine by categorizing quantitative spatial data on CD8+ T cells into a functional and clinically relevant immune phenotype and may become an additional predictive immuno-oncology tool, alone or in combination with established biomarkers.

## Supplementary information

Supplemental Material

## Data Availability

There is an embargo on the data until the final study results are published. Once this is done, the data will be publicly available.
