# Correction: Establishing standardized immune phenotyping of metastatic melanoma by digital pathology

**DOI:** 10.1038/s41374-021-00676-5

**Published:** 2021-09-28

**Authors:** Bettina Sobottka, Marta Nowak, Anja Laura Frei, Martina Haberecker, Samuel Merki, Tumor Profiler Consortium, Mitchell P. Levesque, Reinhard Dummer, Holger Moch, Viktor Hendrik Koelzer

**Affiliations:** 1 Department of Pathology and Molecular Pathology, University and University Hospital Zurich, Zurich, Switzerland; 2 Department of Dermatology, University and University Hospital Zurich, Zurich, Switzerland

**Keywords:** Imaging the immune system, Melanoma, Predictive markers

Correction to: *Laboratory Investigation* 10.1038/s41374-021-00653-y, published online 26 August 2021

The original version of this article unfortunately contained a mistake. The Tumor Profiler Consortium was published as Supplementary Information and was therefore not indexed by PubMed. The error has now been corrected.

