# Supplementary material for: Establishing standardized immune phenotyping of metastatic melanoma by digital pathology
Source: Lab Invest. 2021 Aug 26;101(12):1561–70. doi: 10.1038/s41374-021-00653-y (PMC8590976; doi:10.1038/s41374-021-00653-y)
Supplement: Supplementary file 1 — Supplemental Material [file 41374_2021_653_MOESM1_ESM.pdf]

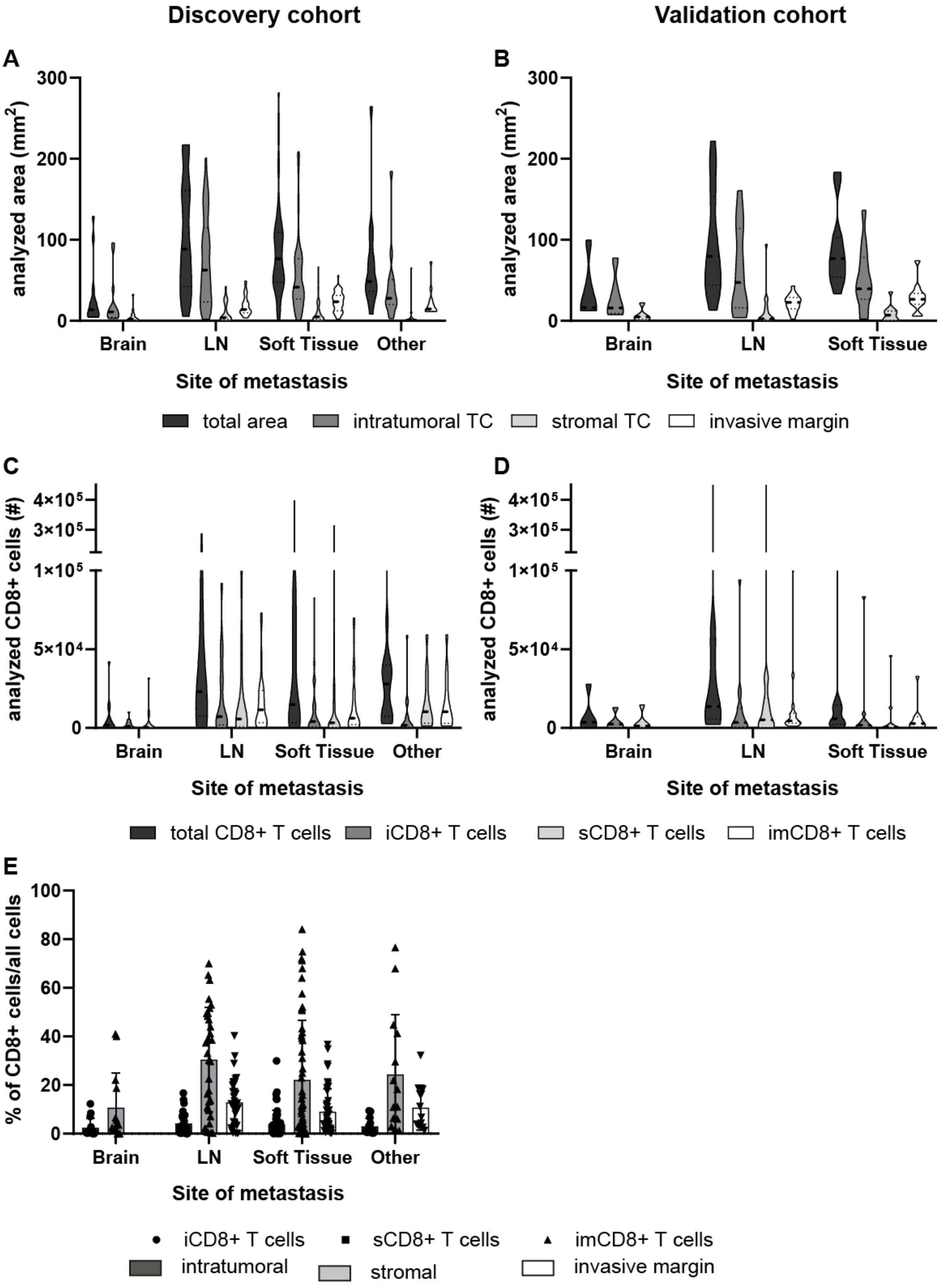
Discovery cohort
Validation cohort
A
B
300
200
100
0
analyzed area (mm2)
Brain
LN
Soft Tissue
Other
Site of metastasis
total area
intratumoral TC
stromal TC
invasive margin
C
D
4×10⁵
3×10⁵
1×10⁵
5×10⁴
analyzed CD8+ cells (#)
total CD8+ T cells
iCD8+ T cells
sCD8+ T cells
imCD8+ T cells
E
100
80
60
40
20
% of CD8+ cells/all cells
iCD8+ T cells
sCD8+ T cells
imCD8+ T cells
intratumoral
stromal
invasive margin

1

**Supplemental Figure 1**

Digital assessment of whole slides depicts the area sizes and amounts of CD8+ T cells analyzed: Areas (**A, B**) and CD8+ T cells (**C, D**) analyzed in total and per respective tumor compartment within the anatomic sites of metastases with comparable results between the discovery cohort (**A, C**) and the validation cohort (**B, D**). An average tissue area of 73 $mm^2$ (range: 32 $mm^2$ (average area brain samples) - 100 $mm^2$ (average area lymph node samples)) was analyzed per case and an average of $3.3 \times 10^4$ CD8+ T-cells (range: $6.8 \times 10^3$ (average CD8+ cells brain samples) - $6.5 \times 10^4$ (average CD8+ cells lymph node samples)) were detected and classified for immune phenotyping. Similar percentages of CD8+ T cells in relation to total cells were detected per tumor compartment irrespective of the metastatic site (**E**). Note that the intratumoral compartment contributes the most to the total analyzed areas or cells whereas the stromal and invasive margin compartment represent only a minor portion.

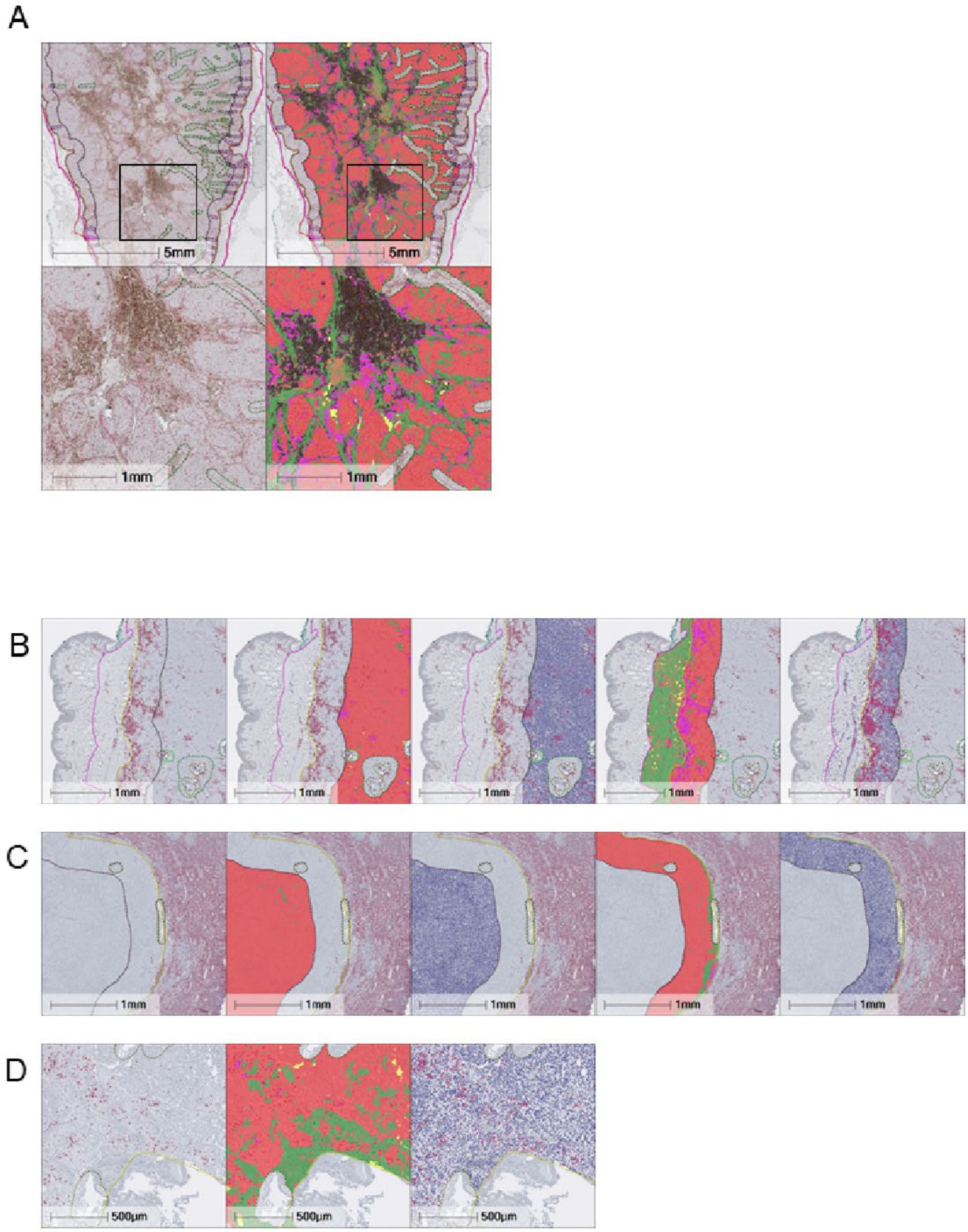


**Supplemental Figure 2**

(**A**) AI-based tissue classification of melanoma metastases into tumor (red), inflamed stroma (purple) and desmoplastic stroma (green); exclusion of glass background

(yellow), melanin pigment (black), hemorrhage and necrosis (orange). (**B**, **C**, **D**) Pathologist guided annotation of the tumor center and invasive margin in melanoma metastases in soft tissue (**B**), lymph node (**C**) and brain (**D**). AI-based tissue segmentation into tumor (red), inflamed stroma (purple) and desmoplastic stroma (green) in tumor center (**B-D**, second panel) and invasive margin (**B-C**, fourth panel). Cell segmentation and scoring at single cell resolution evaluating CD8+ infiltration per $\mu m^2$ in tumor center (**B-D**, third panel) and invasive margin (**B-C**, fifth panel).

| **Clinicopathological Parameters patients (n=28)** | **Site of Metastasis (n=33)** | | | | | |
|---|---|---|---|---|---|---|
| | **Brain (n=5)** | | **Soft tissue (n=11)** | | **LN (n=17)** | |
| | No. | % | No. | % | No. | % |
| **Gender** | | | | | | |
| F | 0 | 0 | 5 | 50 | 7 | 54 |
| M | 5 | 100 | 5 | 50 | 6 | 46 |
| **Age (years)** | | | | | | |
| 40-49 | 1 | 20 | 0 | 0 | 1 | 7 |
| 50-59 | 2 | 40 | 2 | 20 | 4 | 31 |
| 60-69 | 0 | 0 | 1 | 10 | 2 | 14 |
| 70-79 | 1 | 20 | 4 | 40 | 5 | 38 |
| ≥80 | 1 | 20 | 3 | 30 | 1 | 7 |
| **Histological Subtype** | | | | | | |
| Cutaneous | 5 | 100 | 9 | 90 | 8 | 62 |
| Mucosal | 0 | 0 | 0 | 0 | 2 | 14 |
| Ocular | 0 | 0 | 0 | 0 | 1 | 7 |
| Unknown primary | 0 | 0 | 1 | 10 | 2 | 14 |
| **Stage** | | | | | | |
| III | 0 | 0 | 2 | 20 | 3 | 21 |
| IV | 5 | 100 | 6 | 80 | 8 | 62 |
| n.a. | 0 | 0 | 2 | 20 | 2 | 14 |

| | | | | | | |
|---|---|---|---|---|---|---|
| **Immune diagnosis** | | | | | | |
| Desert | 1 | 20 | 3 | 27 | 5 | 30 |
| Excluded | 3 | 60 | 7 | 64 | 9 | 53 |
| Inflamed | 1 | 20 | 1 | 9 | 3 | 17 |

**Supplemental Table 1**

Validation cohort: Clinicopathological data of a retrospective melanoma cohort used as the validation cohort. 33 samples from different anatomical metastatic sites were collected from 28 patients.

**Supplemental Table 2**

**A**

| **Mean CD8+ T cells/μm²** | | **Immune phenotypes** | | |
|---|---|---|---|---|
| | | **desert** (n=10) | **excluded** (n=18) | **inflamed** (n=5) |
| **Tumor compartments** | **iCD8+** | 0.00002 | 0.0001 | 0.0005 |
| | **sCD8+** | 0.0005 | 0.001 | 0.004 |
| | **imCD8+** | 0.001 | 0.001 | 0.003 |

**B**

| ***p* values** | | **Immune phenotypes** | | |
|---|---|---|---|---|
| | | **desert vs excluded** | **desert vs inflamed** | **excluded vs inflamed** |
| **Tumor compartments** | **iCD8+** | n.s. | n.s. | n.s. |
| | **sCD8+** | n.s. | <0.001 | <0.0001 |
| | **imCD8+** | n.s. | <0.01 | <0.05 |

**Supplemental Table 2**

Validation cohort: Means of CD8+ T cell densities according to their spatial distribution and immune phenotype within the validation cohort (A) and the corresponding *p* values using the Holm-Šídák method for multiple comparisons (B). Invasive margin (im) CD8+ for desert n=8, excluded n=15 and inflamed n=4.
